# Long Term Outcome and Histologic Findings of a Retinal Astrocytic Hamartoma Treated with Intravitreal Injection of Anti-VEGF: A Case Report

**DOI:** 10.1155/2021/7500791

**Published:** 2021-09-23

**Authors:** Kinza T. Ahmad, Hana A. Mansour, Benjamin T. Rollins, Sergio Pina Oviedo, Paul H. Phillips, Sami H. Uwaydat

**Affiliations:** ^1^Jones Eye Institute, University of Arkansas for Medical Sciences, AR, USA; ^2^American University of Beirut, Beirut, Lebanon

## Abstract

**Background:**

To our knowledge, this is the first report to describe the histologic changes of a retinal astrocytic hamartoma (RAH) in a patient with tuberous sclerosis complex (TSC) treated with antivascular endothelial growth factor (anti-VEGF), as well as the longest anti-VEGF treatment that such a patient has received (3 years). *Case Presentation*. We present a case of a 20-year-old female with TSC who developed progressive growth of a papillary astrocytic hamartoma that caused significant retinal edema, vitreous hemorrhage, and neovascular glaucoma. The patient was initially treated with 25 intravitreal anti-VEGF injections about every 1-3 months, but eventually developed a blind painful eye from neovascular glaucoma. Histopathologic evaluation of the enucleated globe showed a peculiar difference of the tumor according to its topography, with features reminiscent of pilocytic astrocytoma at the optic nerve head and features reminiscent of subependymal giant cell astrocytoma at the retrolaminar optic nerve. We hypothesize that these changes occurred as a secondary effect of the anti-VEGF treatment.

**Conclusions:**

Anti-VEGF agents may decrease the ophthalmologic complications of RAH. We recommend that this treatment should be started early and continued for a protracted time at regular and frequent intervals. Moreover, a combination of therapies might prove to be superior to monotherapy and should therefore be considered in aggressive retinal astrocytic hamartomas.

## 1. Introduction

Retinal astrocytic hamartomas (RAH) are benign stationary tumors commonly associated with tuberous sclerosis complex (TSC). Tuberous sclerosis is an autosomal dominant, systemic neurocutaneous disorder characterized by proliferation of hamartias and hamartomas in various body parts such as the skin, heart, brain, retina, lungs, and kidneys. About half of patients with TSC have RAH in one or both eyes, which manifest as exophytic or endophytic retinal masses, often multifocal, with involvement of the optic nerve, ciliary body, or iris [1]. These lesions are usually small in size, asymptomatic, and rarely enlarge and may even regress later in life [2]. However, some RAH does enlarge causing complications such as cystoid macular edema, serous retinal detachment, neovascular glaucoma (NVG), or vitreous hemorrhage (VH) [3]. We present the case of a patient, who we have previously reported on, with an optic disc tuber that initially showed signs of enlargement with preservation of vision [4]. Herein, we report the patient's subsequent clinical course, when aggressive enlargement of the RAH resulted in the eventual enucleation of the eye, despite treatment with anti-VEGF. We highlight the patient's clinical course and the histopathologic findings of the enucleated eye and suggest alternatives in the management of similar future patients based on our experience with this patient, as well as a comprehensive review of the literature. To our knowledge, this is the first report that describes the histologic changes of a RAH treated with anti-VEGF, as well as the longest anti-VEGF treatment that a patient with RAH has received, spanning a total of three years.

## 2. Case Presentation

**Figure 1:**
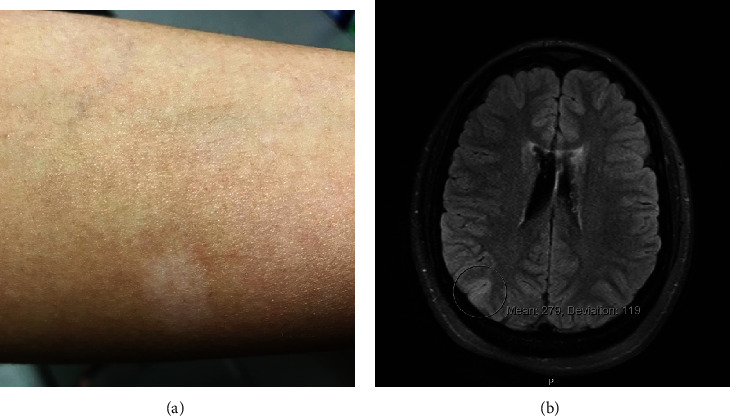
(a) External color photograph demonstrating a cutaneous ash leaf spot on the patient's forearm. (b) MRI of the brain showing FLAIR hyperintensities involving the right posterior parietal lobe demonstrating a cortical tuber (circled).

**Figure 2:**
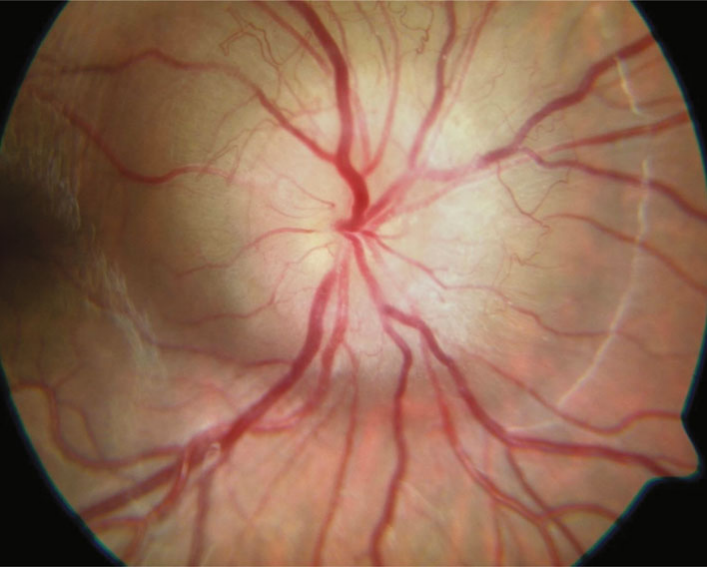
Fundus exam at 12 years of age of the right eye showing significant elevation of the optic disc from peripapillary solid infiltration of the retina.

**Figure 3:**
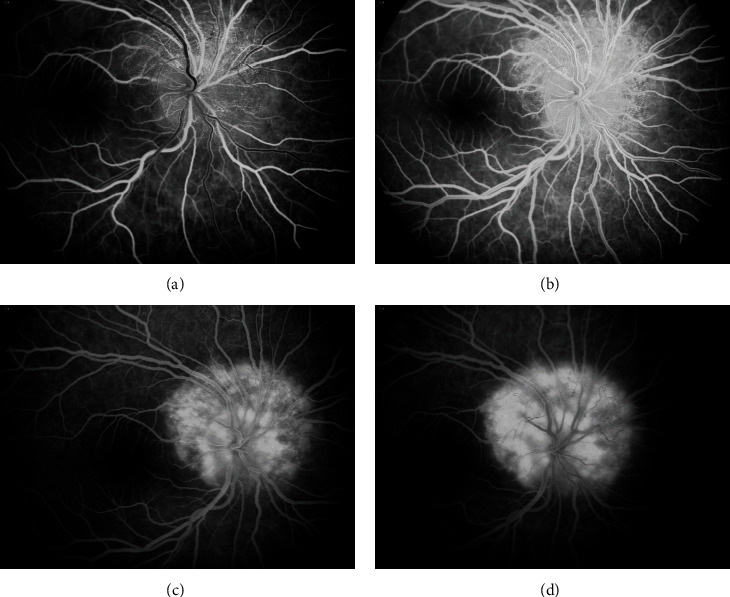
Fluorescein angiogram of the right eye at 12 years of age. (a) Early phase shows filling of the arteries and laminar flow in the veins. (b) Arteriovenous phase reveals filling of a fine network of “filigree” vessels around the optic nerve head. (c) and (d) The late phases show diffuse hyperfluorescence and nodular staining of the tumor.

**Figure 4:**
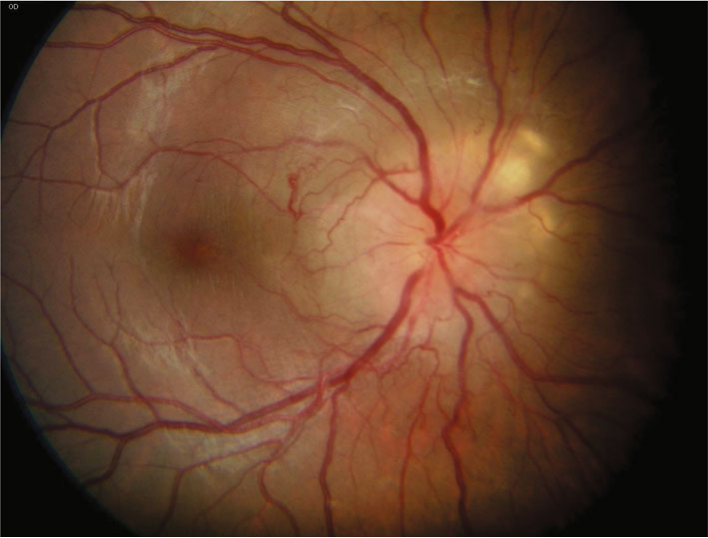
Fundus exam at 15 years of age of the right eye continues to show significant elevation of the optic disc with peripapillary exudation and peripapillary hemorrhages from the filigree vessels.

**Figure 5:**
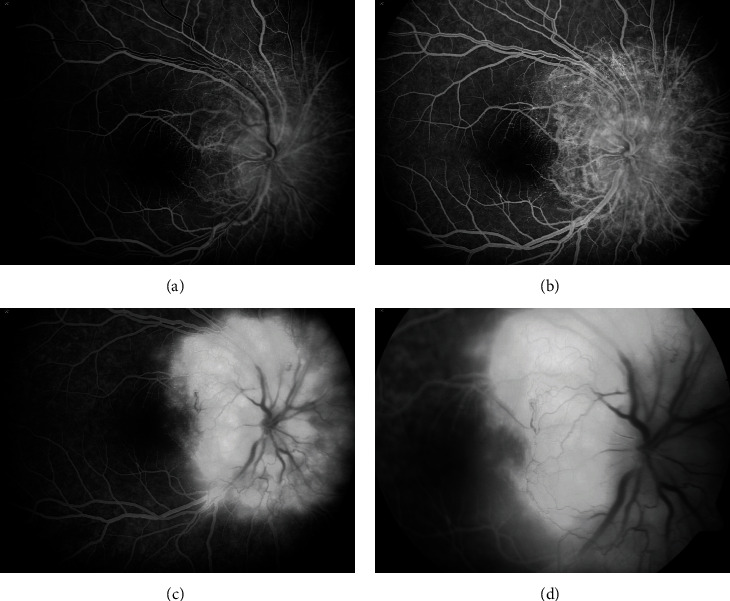
At age 15, fluorescein angiogram of the right eye shows extensive peripapillary leakage in the late phase (c) and (d) from the filigree vascular network of the tumor with fluid extending into the macula.

**Figure 6:**
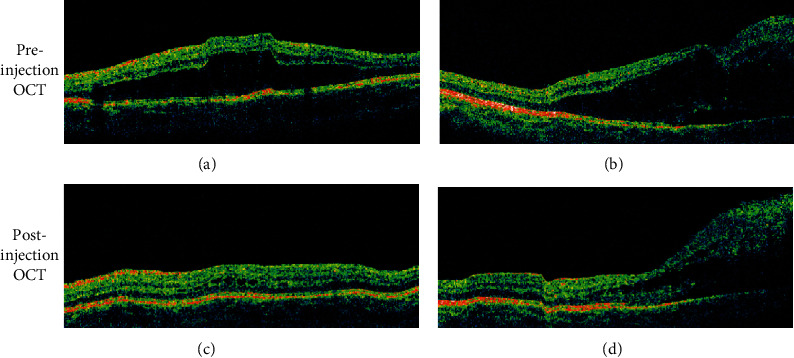
Preinjection (a) and (b) and postinjection (c) and (d) optical coherence tomography (OCT) photos of the right eye over the macula (a) and (c) and nerve (b) and (d). The preinjection OCT photo over the nerve (b) shows the optic disc tumor with peripapillary subretinal fluid extending to the macula (a). Significant improvement in subretinal fluid is seen after intravitreal injection of anti-VEGF (c) and (d). Despite improvement on the OCT, the vision remained stable at 20/200.

A 20-year-old Caucasian woman received the diagnosis of TSC at an early age following seizure attacks with findings of multiple central nervous system subependymal nodules, cortical tubers, cutaneous ash leaf spots (Figure 1), and facial angiofibromas. Subsequent genetic testing revealed that she was heterozygous for a frameshift mutation in *TSC1*. At the age of 19 months, the patient had a swollen right optic disc along with a relative afferent pupillary defect in the right eye. At 12 years of age, visual acuity was 20/20 bilaterally with normal color vision testing. By then, the right optic disc was severely elevated with peripapillary solid infiltration of the retina and an overlying lace-like network of “filigree” vessels (Figure 2) well-illustrated on fluorescein angiography (Figure 3). At this point, the patient was followed periodically. Visual acuity in the right eye gradually dropped to 20/25 at age 14, then 20/60, and subsequently to 20/80 at age 15. At this time, fundus exam showed significant elevation of the optic disc with peripapillary exudation and hemorrhages from the filigree vessels (Figures 4 and 5) with serous macular elevation on spectral domain optical coherence tomography (SD-OCT) (Figure 6). After a thorough discussion of the available therapeutic options, the patient and family are elected to proceed with an off-label use of intravitreal bevacizumab (1.25 mg in 0.05 mL). Monthly injections were initiated and the subretinal macular fluid improved after each injection; however, the patient only noted subjective visual improvement after the injections and her vision eventually stabilized at 20/200. When the interval between the anti-VEGF injections was increased to 8-12 weeks, the patient developed VH and was returned to a 4-7-week injection regimen. The filiform vessels continued to extend with the relentless growth of the peripapillary retinal mass. After 9 intravitreal injections, the patient was lost to follow-up for 10 months. Subsequently, she presented with another VH and received thereafter ranibizumab (2 mg in 0.05 mL). When she missed her monthly injection, VH would recur. The macular edema became unresponsive to the injections with visual acuity maintaining at 20/400. After a total of 16 ranibizumab injections, she missed her follow-up visit and presented 10 months later with HM vision, dense VH, and NVG, for which a pars plana vitrectomy (PPV) with panretinal photocoagulation and Ahmed tube placement was done. Five months later, her vision had dropped to light perception, with persistent VH and NVG. She underwent a repeat PPV with phacoemulsification cataract surgery and tube revision. Postoperatively, her vision dropped to no light perception (NLP), and five months later, with persistent NLP vision and a painful eye, the patient made the decision to proceed with enucleation.

### 2.1. Pathologic Findings

**Figure 7:**
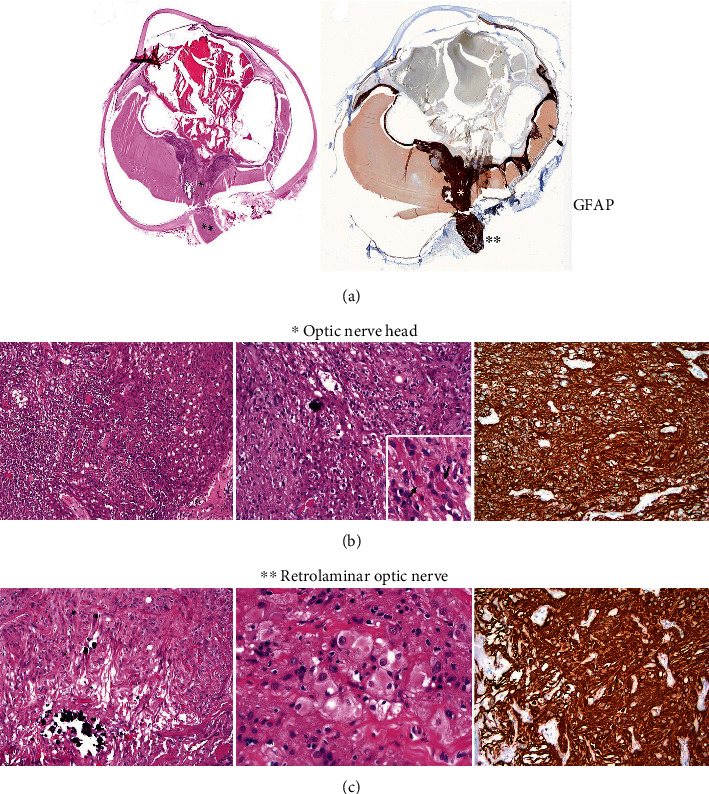
(a) Full montage of the enucleation specimen. (Left) The right eye showed complete serosanguinous retinal detachment, ectropion uveae, and anterior and posterior synechiae. The optic nerve head (∗) is expanded and hypercellular with focal calcifications. In this image, the retrolaminar optic nerve appears normal (∗∗) (hematoxylin and eosin stain). (Right) These areas are positive for glial fibrillary acidic protein (GFAP). (b) Astrocytic proliferation in the optic nerve head reminiscent of pilocytic astroctyoma. (Left panel) Areas of necrosis and prominent neovascularization are seen (10×). (Middle panel) Scattered calcifications throughout the lesional tissue (20×). Few eosinophilic granular bodies (arrows) are seen (40×). (Right panel) Immunohistochemistry for glial fibrillary acidic protein (GFAP) highlights the astrocytic proliferation (40×) (Left and middle panels: hematoxylin and eosin stain). (c) Astrocytic proliferation in the retrolaminar optic nerve reminiscent of subependymal giant cell astrocytoma. (Left panel) Large eosinophilic polygonal cells involving the optic nerve. There are focal calcifications (10×). (Middle panel) These large cells were located in between nerve fibers forming cords or distributed as single cells throughout the retrolaminar optic nerve. At higher magnification, the peripheral vacuolization and plump, granular, and eosinophilic cytoplasm of these cells is apparent. The nuclei and nucleoli are readily visible with some cells displaying irregular nuclear contours. (Right panel) Immunohistochemistry for glial fibrillary acidic protein (GFAP) is positive in these large epithelioid cells supporting a glial/astrocytic origin (20×) ((a)–(c): hematoxylin and eosin stain).

Examination of the enucleated specimen revealed the presence of a very vascular hamartoma of the optic nerve and surrounding retina with superficial vessels corresponding to the filiform vessels. On microscopic examination, histologic sections showed an astrocytic proliferation at the optic nerve (Figure 7(a)) that differed as to location within the nerve. At the optic nerve head and adjacent retina, the astrocytic proliferation showed a fibrillary background with a variable number of microcysts (Figure 7(b), left panel). Astrocytes showed spindle morphology with long slender cytoplasmic processes and hyperchromatic, enlarged, and elongated to slightly irregular nuclei. Rare mitoses were seen. Focal calcifications were present (Figure 7(b), middle panel). At higher magnification, occasional eosinophilic granular bodies were seen (Figure 7(b), middle panel inset). All these features were reminiscent of those seen in pilocytic astrocytoma. In addition, foci of subretinal necrosis with acute inflammation and extensive preretinal neovascularization were observed (Figure 7(b), left and middle panels). In contrast, the retrolaminar optic nerve showed a proliferation of large polygonal cells with abundant eosinophilic cytoplasm with variable vacuolization, oval to round nucleus, and prominent nucleolus that diffusely infiltrated through the nerve fibers (Figure 7(c), left and middle panels). Focal calcifications were also identified (Figure 7(c), left panel). The features at the retrolaminar optic nerve were similar to those of subependymal giant cell astrocytoma (SEGA). By immunohistochemistry, the astrocytic proliferation at both the optic nerve head (Figure 7(b), right panel) and retrolaminar area (Figure 7(c), right panel) was diffusely and strongly positive for glial fibrillary acidic protein (GFAP). Ki-67 was low (2-3%) at both sites (not shown), demonstrating the lower proliferative rate of the tumor.

## 3. Discussion

Our case highlights the course of a clinically aggressive papillary astrocytic hamartoma causing macular exudative detachment that was initially controlled with intraocular injections of anti-VEGF over the course of three years. The patient was periodically lost to follow-up, and the injections were inconsistent. Despite PPV for recurrent VH and a glaucoma tube for NVG, the eye became blind and painful and was ultimately enucleated.

Tuberous sclerosis-associated retinal lesions have been classified into 3 morphological groups [5]: type 1 is an oval flat light-grey smooth lesion in the retinal nerve fiber layer measuring a half-disc diameter; type 2 is a mulberry calcified lesion, usually multiple in number, in the posterior pole; and type 3 is a mixture of type 1 and 2 lesions, consisting of a calcific center with a smooth perimeter. A previous study found that symptomatic progression is most commonly seen in type 1 lesions, while only 25% of type 2 lesions were noted to progress [5]. Shields et al. reported on four patients with TSC and RAH with aggressive growth and found that TSC lesions near the optic disc tend to grow while lesions farther from the disc tended to be stationary [6]. Our case is of a type 1 tumor, arising from the optic nerve and demonstrating aggressive growth during our patient's teenage years. Retinal hamartomas that exhibit an aggressive growth are often composed of SEGA-like cells [6]. Previous reports have described the use of argon laser and photodynamic therapy to treat RAH associated with retinal exudates [1, 7]. The location of the lesion in our patient, however, precluded the use of these modalities. Recently, VEGF has been shown to be an important angiogenic factor in malignant astrocytomas of the brain [8, 9], and it has also been detected in retinal astrocytomas [3]. The hypothesized mechanism for the effect of anti-VEGF agents is the limitation of tumor growth and decreased leakage from the preexisting rich vascular network that characterizes RAH lesions. An additional theory supporting the use of VEGF blockade is the genetic nature of TSC. Tuberous sclerosis complex results from a mutation in either one of two unlinked genes, *TSC1* or *TSC2*. Both *TSC1* and *TSC2* are tumor suppressor genes with their respective protein products hamartin and tuberin regulating cellular proliferation, adhesion, growth, differentiation, and migration [10]. *TSC1* or *TSC2* mutations and loss of their protein products result in the accumulation of hypoxia-inducible factor-1*α* (HIF-1*α*) and increased expression of HIF-responsive genes including VEGF [11]. VEGF antagonists have been used in several patients with RAH but the follow-up was short, and the studies reported between 1 and 6 total injections [12, 13]. Other reported therapies have included dexamethasone implants, corticosteroid injections, systemic sirolimus or everolimus, photodynamic therapy, laser photocoagulation, and vitrectomy [7, 14–18]. Saito et al. published a study in 2010 where they treated two patients with RAH with intravitreal bevacizumab with resolution of leakage related to the hamartoma [13]. The first patient had previously undergone a vitrectomy for nonresolving VH with laser to the retinal tumors, and the second patient received one injection but was followed only for 3 months. In 2012, Tomida et al. described a case of a young man with aggressive RAH due to TSC who received monthly intravitreal bevacizumab injections (1.25 mg) for a total of 6 injections [3]. Two months after the sixth injection, enucleation was done for severe VH. In that case, the RAH showed immunoreactivity for VEGF and the overlying epiretinal membrane demonstrated coexpression of both VEGF and von Willebrand factor (VWF), indicating the presence of VEGF in the vascular endothelial cells. In contrast to the high VEGF immunoreactivity in the tumor, the VEGF level in the vitreous was undetectable by enzyme-linked immunosorbent assay. More recently, Rajasekaran in 2019 published a report on a patient that had a 3-line improvement in vision 2 weeks after a single injection of bevacizumab for a lesion with significant serous fluid [12].

In our patient, after an initial response to VEGF antagonists, both exudative and hemorrhagic complications were subsequently poorly responsive. The poor response can partially be attributed to the natural history of progressive growth of such tumors at the nerve head and also to the intermittent cessation of VEGF blockade resulting in rebound VEGF overexpression. Our case also suggests that intravitreal injection of anti-VEGF agents suppresses vitreous VEGF and also inhibits VEGF activity in certain anterior portions of the tumor in contact with the vitreous cavity. This is supported by our finding of only pilocytic cells in the prelaminar space, compared to predominantly SEGA-like astrocytic cells in the retrolaminar space. Typically, RAH lesions have components of both pilocytic and SEGA-like cells[6], but previous studies have not specified a preferential distribution of these cells as seen in our case, nor have they documented any cases with only one tumor component. Prior histopathologic studies have shown that RAH in the setting of TS not treated with anti-VEGF medications demonstrates variable proportions of SEGA-like cells and spindle cells, with SEGA-like cells invariably present in both retinal and ON compartments in all cases [6]. To the best of our knowledge, no reports of a specific compartmentalization of these morphologic patterns—as seen in our case—have been described. Based on these prior observations, we hypothesize that, in our patient, the SEGA-like cells might have initially been present at both retrolaminar and prelaminar sites, but the anti-VEGF treatment might have affected the morphology of the SEGA-like cells preferentially at the prelaminar site leaving only the pilocytic astrocytoma-like cells at the prelaminar optic nerve.

Additionally, our findings agree with other studies that have shown the macular edema and neovascularization in TSC can recur despite intravitreal bevacizumab monotherapy [3]. The clinical course and histochemical findings in our patient indicated that intravitreal VEGF blockade may have been insufficient to treat such an aggressive RAH and a more regular monthly regimen of injections combined with other modalities (laser photocoagulation, photodynamic therapy, dexamethasone implant, sirolimus, or everolimus) could have been considered.

## 4. Conclusion

In conclusion, this case suggests that VEGF antagonism can change the morphology of the more aggressive SEGA-like cellular component of RAH in the setting of TSC. Additionally, anti-VEGF agents may decrease the ophthalmic complications of TSC and should be started early and continued for a protracted time at regular and frequent intervals. Discontinuation of VEGF antagonism can lead to VEGF overexpression and loss of therapeutic effect. A combination of therapies (laser photocoagulation, photodynamic therapy, dexamethasone implant, sirolimus or everolimus, and intravitreal VEGF antagonists) might prove to be superior to monotherapy and should therefore be considered, especially in aggressive RAH.

## Data Availability

All data generated or analyzed during this study are included in this published article.

## Abbreviations

RAH: Retinal astrocytic hamartoma
TSC: Tuberous sclerosis complex
NVG: Neovascular glaucoma
VH: Vitreous hemorrhage
PPV: Pars plana vitrectomy
NLP: No light perception
SEGA: Subependymal giant cell astrocytoma
GFAP: Glial fibrillary acidic protein
HIF-1*α*: Hypoxia inducible factor-1*α*.

## References

[1] Yan S., Chen Y., Chen R., Tian B., Li Z. (2018). Subthreshold micropulse laser photocoagulation therapy in a case of bilateral retinal astrocytic hamartomas with tuberous sclerosis complex. *Medicine*.

[2] Zimmer-Galler I. E., Robertson D. M. (1995). Long-term observation of retinal lesions in tuberous sclerosis. *American journal of ophthalmology.*.

[3] Tomida M., Mitamura Y., Katome T., Eguchi H., Naito T., Harada T. (2012). Aggressive retinal astrocytoma associated with tuberous sclerosis. *Clinical Ophthalmology*.

[4] Brodsky M. C., Safar A. N. (2007). Optic disc tuber. *Archives of Ophthalmology*.

[5] Mennel S., Meyer C. H., Peter S., Schmidt J. C., Kroll P. (2007). Current treatment modalities for exudative retinal hamartomas secondary to tuberous sclerosis: review of the literature. *Acta Ophthalmologica Scandinavica.*.

[6] Shields J. A., Eagle R. C., Shields C. L., Marr B. P. (2005). Aggressive retinal astrocytomas in 4 patients with tuberous sclerosis complex. *Archives of Ophthalmology*.

[7] Mennel S., Hausmann N., Meyer C. H., Peter S. (2006). Photodynamic therapy for exudative hamartoma in tuberous sclerosis. *Archives of Ophthalmology*.

[8] Oehring R. D., Miletic M., Valter M. M. (1999). Vascular endothelial growth factor (VEGF) in astrocytic gliomas–a prognostic factor?. *Journal of neuro-oncology.*.

[9] Pietsch T., Valter M. M., Wolf H. K. (1997). Expression and distribution of vascular endothelial growth factor protein in human brain tumors. *Acta Neuropathologica*.

[10] Rosset C., Netto C. B., Ashton-Prolla P. (2017). TSC1 and TSC2 gene mutations and their implications for treatment in tuberous sclerosis complex: a review. *Genetics and Molecular Biology*.

[11] Brugarolas J. B., Vazquez F., Reddy A., Sellers W. R., Kaelin W. G. (2003). TSC2 regulates VEGF through mTOR-dependent and -independent pathways. *Cancer Cell*.

[12] Rajasekaran N. M., Horo S., Kuriakose T. (2019). Primary ocular presentation of tuberous sclerosis–a case report. *Indian Journal of Ophthalmology*.

[13] Saito W., Kase S., Ohgami K., Mori S., Ohno S. (2010). Intravitreal anti-vascular endothelial growth factor therapy with bevacizumab for tuberous sclerosis with macular oedema. *Acta ophthalmologica*.

[14] Eskelin S., Tommila P., Palosaari T., Kivelä T. (2008). Photodynamic therapy with verteporfin to induce regression of aggressive retinal astrocytomas. *Acta Ophthalmologica*.

[15] Tang P. L., Bastion M. L. (2015). Tuberous sclerosis presenting as atypical aggressive retinal astrocytoma with proliferative retinopathy and vitreous haemorrhage. *Brunei International Medical Journal*.

[16] Bloom S. M., Mahl C. F. (1991). Photocoagulation for serous detachment of the macula secondary to retinal astrocytoma. *Retina*.

[17] Nemoto E., Morishita S., Akashi M. (2017). A case of proliferative retinopathy complicated with tuberous sclerosis treated by vitreous surgery. *Case Reports in Ophthalmology*.

[18] Nakayama M., Keino H., Hirakata A., Okada A. A., Terado Y. (2012). Exudative retinal astrocytic hamartoma diagnosed and treated with pars plana vitrectomy and intravitreal bevacizumab. *Eye*.

